# Preparation of Continuous Highly Hydrophobic Pure Silica ITQ-29 Zeolite Layers on Alumina Supports

**DOI:** 10.3390/molecules25184150

**Published:** 2020-09-10

**Authors:** Miguel Palomino, Hideki Ono, Susana Valencia, Avelino Corma

**Affiliations:** 1 Instituto de Tecnología Química (UPV-CSIC), Universitat Politècnica de València, Consejo Superior de Investigaciones Científicas, Av. de los Naranjos s/n, 46022 Valencia, Spain; miparo@itq.upv.es; 2 Central Technical Research Laboratory, ENEOS Corporation, 8, Chidoricho, Naka-ku, Yokohama 231-0815, Japan; ono.hideki@eneos.com

**Keywords:** ITQ-29 zeolite, LTA structure, pure silica, zeolite membrane

## Abstract

The preparation of continuous layers of highly hydrophobic pure silica ITQ-29 zeolite, potentially applicable as hydrophobic membranes for separation of molecules based on their polarity, has been investigated. Continuous layers of intergrown ITQ-29 zeolite crystals were successfully grown on porous alumina supports by optimization of the synthesis conditions, such as the appropriate selection of the seeds, the procedure for the gel preparation, and the calcination conditions. This resulted in the formation of all silica ITQ-29 zeolite layers without the presence of germanium required in previously reported ITQ-29 membranes, with the subsequent improvement in quality and stability, as verified by the absence of cracks after calcination. We have proved that the incorporation of aluminum from the support into the zeolite layer does not occur, neither during the secondary growth nor through migration of aluminum species during calcination.

## 1. Introduction

The production of platform molecules from renewable sources has been pointed out as an alternative to the use of petroleum due to the growing interest on low-carbon technologies. Butanol arises as a very versatile molecule, not only as excellent biofuel, but also as a reactant for the preparation of important industrial chemicals [1]. When n-butanol is produced biologically from fermentation of starchy and sugar feedstocks, through the process called ABE (acetone-butanol-ethanol), this alcohol is known as biobutanol, and can be produced by a diversity of different micro-organisms from the Clostridiaceae bacterial family [2]. The ABE fermentation typically results in a 3:6:1 ratio of acetone, butanol, and ethanol, with a total product concentration of 2–3 wt.% in water, resulting in a butanol concentration of 12 g/L [3]. The separation of the diluted biobutanol from the aqueous solution is very costly by conventional distillation processes, even surpassing the energy content of the butanol itself [4]. Therefore, alternative and more efficient separation methods have been studied, and those based on adsorption processes have been pointed out as competitive and economically viable [5]. The studied adsorbents comprises different kind of materials, such as activated carbons, zeolites, metal–organic frameworks (MOFs) and polymeric resins [6,7,8,9]. Zeolites arise as one of the most versatile microporous materials used in the separation field due to their tunable properties, like their pore size, geometry and distribution, thermal and chemical stability, polarity and nature, among others [10].

Zeolites are crystalline microporous aluminosilicates in which the silicon and aluminum tetrahedra, SiO_4_ and AlO_4_, are linked by sharing oxygen atoms and assembled into secondary building units such as cubes, hexagonal prisms, octahedra, etc., generating a vast variety of microporous structures accessible through different types and sizes of windows to pores and cavities of molecular dimensions. At present, there are more than 260 different zeolitic structures, as it is reported in the database of the International Zeolite Association that collects all related information and ascribes a three-letter code to each structure [11]. A pure silica zeolite results in a neutral framework, but the isomorphous substitution of silicon with aluminum atoms yields to negative charges into the framework that are compensated by inorganic or organic extraframework cations (typically alkali, alkylammonium, or protons). This phenomenon provides to zeolites a very wide variety of properties that have been explored in fields of industrial relevance like catalysis, ion exchange, and as selective adsorbents in separation processes [12,13].

Zeolites can also be synthesized in different appearances, crystal sizes, and shapes, being also possible to grow them into a continuous intergrown membrane [14]. There are several methods for the preparation of zeolite films and membranes. The most commonly used methods in the literature are: in situ crystallization, vapor phase transport, and seeding method. The most versatile methodology is the seeding procedure, where a layer of pre-synthesized zeolite crystals is deposited on the surface of the porous support and subsequently submitted to hydrothermal treatment in the presence of the synthesis gel. This approach is known as secondary growth. The zeolite membranes are typically prepared on porous supports, which will confer the required mechanical strength to hold the selective thin layer of intergrown zeolite. A thin film possesses a low mass transfer resistance which is necessary for a high flux membrane. So, a defect-free zeolite membrane will take advantage of its very narrow pore size distribution for molecular sieving in a less energetically demanding continuous process.

Pure silica zeolite membranes have attracted interest for decades because of their hydrophobicity and high thermal and chemical stabilities, and they have proved potential applicability to carry on organic/water separations, being Silicalite-1 zeolite the most studied [15,16,17,18,19]. Some other siliceous zeolite membranes of different crystalline structure have also been recently reported, such as BEA [20], CHA [21], and STT [22].

The polarity of zeolites can be finely tuned by controlling the Si/Al ratio in the framework. For instance, the small pore zeolite with the code LTA (Linde Type A) can be obtained in the whole range of Si/Al ratios, thus covering the widest range of polarity, from the very hydrophilic Si/Al ratio of 1 in zeolite A [23], to the very hydrophobic zeolite with an all silica composition, known as zeolite ITQ-29 [24]. So, their polarity can be modified in order to promote the adsorption and diffusion of selected molecules. ITQ-29 has been proven to be the most hydrophobic of the LTA zeolites, based on the isosteric heat of CO_2_ adsorption, making this zeolite a suitable adsorbent for natural gas upgrading, due to its high regenerability and adsorption capacity of CO_2_ over CH_4_ [25,26]. Also, ITQ-29 has been studied for the isolation of biobutanol from an aqueous acetone–butanol–ethanol (ABE) fermentation media [27], but in this case, using ITQ-29 zeolite in powder or as an extrudate. It was possible to reach a butanol purity of >99.5%, at a recovery of >99% by combining columns of hydrophobic ITQ-29 zeolite and the polar SAPO-34 (silicoaluminophosphate of CHA structure). The hydrophobic character of pure silica ITQ-29 zeolite has been clearly evidenced by the corresponding water adsorption measurements. It was reported that all silica ITQ-29 adsorbed 1 wt.% water at 20 mbar and 298 K compared with 26 wt.% in the case of CaA (same LTA structure with Si/Al ratio of 1 and Ca as charge compensating cations) [24]. More recently, the water adsorption was quantified in 4 wt.% measured at 85% humidity and 313 K, being significantly lower than the capacity of other pure silica zeolite (Chabazite) that adsorbed 8 wt.% under the same conditions [27]. The hydrophobicity of the ITQ-29 zeolite is compared in the present work with that of a very well-known and reported hydrophobic zeolite, such as Silicalite-1 (MFI structure). Silicalite-1 has been reported as a very hydrophobic material, even when compared with other pure silica zeolites, yielding to the lowest affinity to water against ZSM-5, TS-1, and Beta zeolites by TPD techniques [28], vapor adsorption experiments [29] and molecular dynamics [30].

A first approach to the preparation of ITQ-29 membranes was done on porous tubular supports, but using germanium in the synthesis gel, resulting in Ge-ITQ-29 zeolite [31], because germanium directs the formation of double four-rings (D4R), present in the LTA structure [24]. Although the Ge content in the gel for the secondary growth of the membranes was relatively low (Si/Ge molar ratio of 20), it was found that it was preferentially incorporated into the zeolite framework, thus resulting in a lower Si/Ge ratio. Ge-containing zeolites are very sensitive to moisture after calcination, because of the easy hydrolysis of the Ge-O bonds under ambient moisture, with the subsequent loss of crystallinity. Although the membranes were calcined in the presence of ozone, they are prone to suffer crack formation during calcination, even at relatively low temperature, most likely due to differences in the thermal expansion coefficients of the zeolite and that of the tubular support.

The preparation of pure silica ITQ-29 thin films on silicon wafers was also reported via the fluoride-assisted synthesis in vapor phase to produce low dielectric constant materials with potential interest in the semiconductors industry [32]. Finally, another approach to the preparation of membranes based on ITQ-29 was by using the zeolite powder as a filler in mixed matrix membranes for the separation of CO_2_ and N_2_. In this case, LTA zeolites with Si/Al ratio of 5 [33,34] and the pure silica ITQ-29 [35] were used for preparing mixed matrix membranes using poly(1-trimethylsilyl-1-propyne) (PTMSP) as the polymer matrix. It was found that the permselectivity to CO_2_ was considerably improved by increasing the zeolite loading into the polymer membrane. Nevertheless, in the case of mixed matrix membranes the compatibility between the polymer membrane and the selective filler is of crucial importance and the zeolite is unevenly distributed along the membrane.

Therefore, we decided to go one step forward in the preparation of pure-silica ITQ-29 membranes on porous ceramic supports with the objective of preparing continuous pure silica zeolite ITQ-29 layers free of cracks. A key aspect to take into account during the membrane growth is to ensure an appropriate contact between the seeded support and the synthesis gel. It becomes critical when using a solid synthesis gel such as the one used for the standard preparation of ITQ-29 zeolite which is done in fluoride medium from a highly concentrated gel. So, the gel preparation procedure was modified in order to increase the gel viscosity. Besides, we decided to use other type of support and planar alumina discs were selected with the aim of decreasing as much as possible the strains between the zeolite layer and the support during calcination.

In this context, we propose the use of an ITQ-29 zeolite membrane grown on porous alumina supports as an appropriate candidate for the separation of bioalcohols from fermentation because of its very high hydrophobicity.

## 2. Results and Discussion

### 2.1. ITQ-29 Seeds

**Figure 1:**
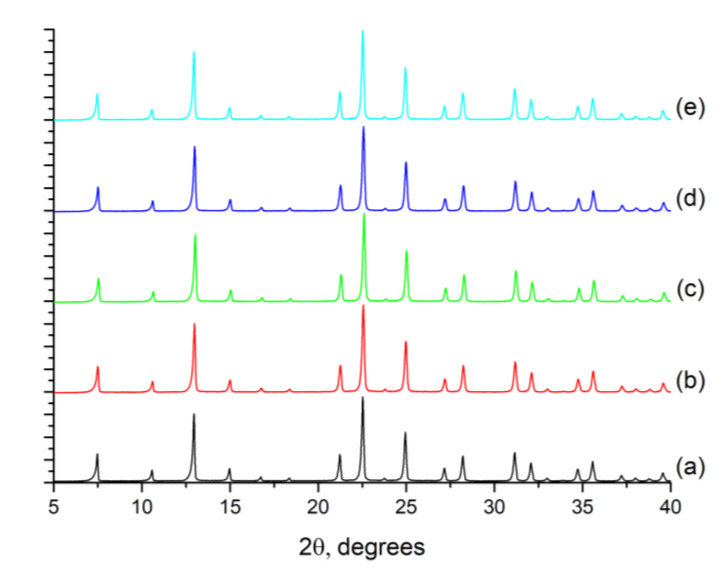
X-ray diffraction patterns of: (**a**) reference ITQ-29 zeolite, prepared as described in [24]; and samples synthesized in this work under different conditions for the use as seeds: (**b**) dynamic synthesis, 3.5 days; (**c**) dynamic synthesis, 4.5 days; (**d**) static synthesis, 3.5 days; (**e**) static synthesis, 4.5 days.

First, the synthesis of the seeds to be used in the preparation of the zeolite layers on the alumina supports was optimized with the aim of controlling the crystal size. Thus, the influence of the synthesis duration and the synthesis procedure (crystallization in static or dynamic conditions) on the size and crystallinity of the ITQ-29 zeolite was studied herein. The purity of the ITQ-29 samples was confirmed by X-ray diffraction (XRD), all of them are pure LTA zeolites with high crystallinity, as can be seen in Figure 1, where the synthesized samples are compared with a reference pure silica ITQ-29 zeolite [24].

**Figure 2:**
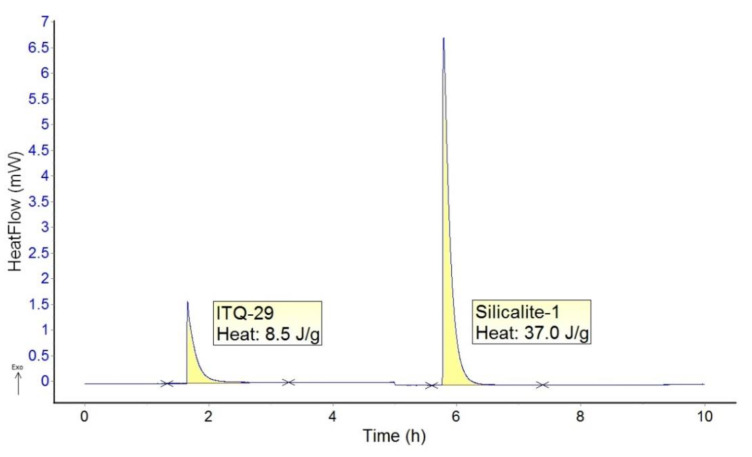
Heat of immersion in water measured by calorimetry at 303 K on ITQ-29 and Silicalite-1.

The hydrophobicity of ITQ-29 (sample shown in Figure 1b) and Silicalite-1 zeolites was compared by immersion calorimetry experiments in water at 303 K. After exposing both zeolites in identical experiments to liquid water, the amount of heat evolved in the case of ITQ-29 is significantly lower than in the case of Silicalite-1. Both experiments are appended together for ease of comparison, yielding values of 8.5 and 37.0 J/g respectively, as shown in Figure 2, resulting in a ratio of 4.4. This is quantified per gram of material, but if the comparison is done by surface area, the results correspond to areal enthalpies of immersion in water of 10 and 82 mJ/m^2^ for ITQ-29 and Silicalite-1, respectively. This fact is a clear indication of the very hydrophobic character of ITQ-29 zeolite, even surpassing that of Silicalite-1, reported as a hydrophobic material. As a comparison, the heat of immersion in water of a hydrophilic material, such as the ordered mesoporous MCM-41, gives a value of 135 mJ/m^2^. The very high hydrophobicity is attributed not only to the lack of charges due to the pure silica composition, but also to the absence of internal silanol defects on the ITQ-29 zeolite [24]. This behavior is encouraging to prepare continuous defect-free membranes with capability to perform separations based on the polarity of the mixture components.

**Figure 3:**
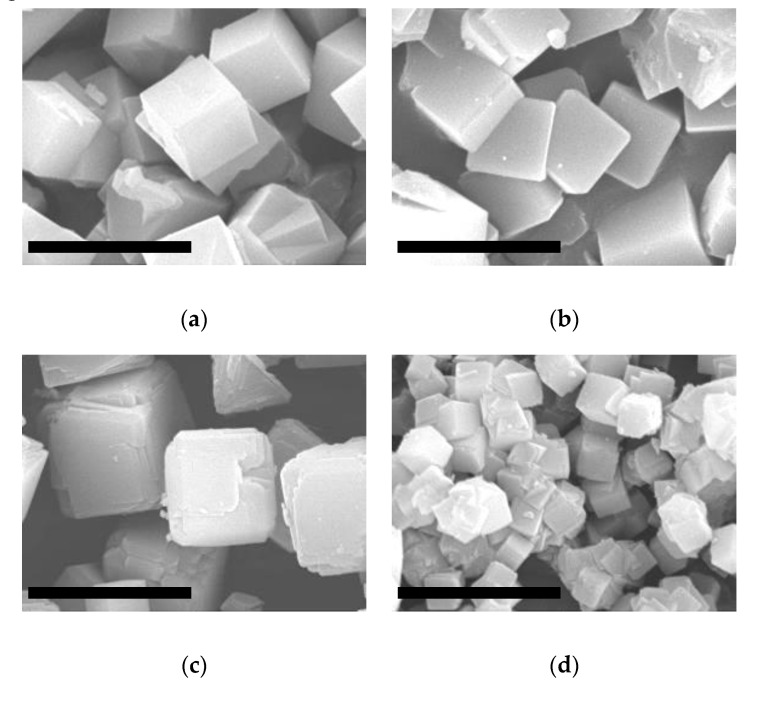
SEM images of ITQ-29 samples synthesized in different conditions: (**a**) static, 4.5 days; (**b**) static, 3.5 days; (**c**) dynamic, 4.5 days; (**d**) dynamic, 3.5 days. Scale bars correspond to 6 µm.

The crystal morphology and size of the crystallites was studied by scanning electron microscopy (SEM) and the images are shown in Figure 3. It was observed that the size of the ITQ-29 crystals increased with the crystallization time and working under static conditions. In all cases, the crystals exhibit the characteristic cubic shape of zeolites with LTA structure. It is worth noting that the effect of the synthesis duration is less pronounced in the case of static conditions, obtaining crystallites of similar size, of about 3 and 4 µm, after 3.5 and 4.5 days respectively. When the dynamic crystallization was used, crystals of 5–6 µm were obtained after 4.5 days of synthesis, but smaller crystallites of about 0.6 µm were synthesized after 3.5 days. So, these small crystallites of regular shape and high crystallinity were selected to be used as seeds during the preparation of the ITQ-29 membranes by secondary growth. The use of smaller crystals for seeding the alumina supports is highly convenient for achieving a good dispersion of the crystallites within the porosity of the alumina, thus favoring the secondary growth of the zeolite membrane.

### 2.2. ITQ-29 Zeolite on Porous Alumina Supports

#### 2.2.1. Seeding of the Supports

**Figure 4:**
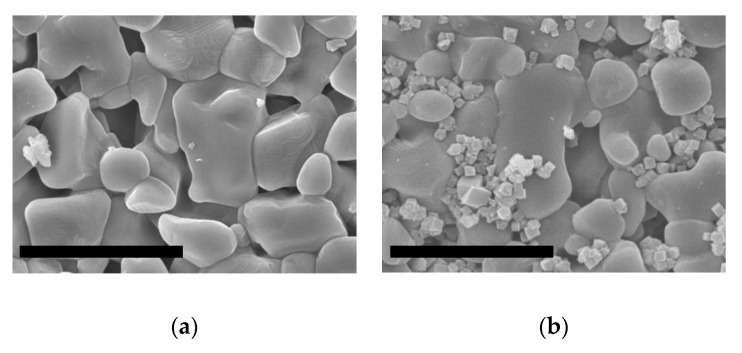
SEM images of alumina supports: (**a**) unseeded; (**b**) seeded with ITQ-29 zeolite. Scale bars correspond to 10 µm.

The alumina porous supports were successfully seeded with an evenly distributed layer of 0.6 µm crystals of ITQ-29 zeolite by using the dip-coating procedure with an aqueous solution of polyethyleneimine. In Figure 4 are shown a bare alumina support and a seeded one, where the brighter crystals corresponding to the seeds can be seen on the surface of the alumina disk. Because of the small size of the crystals, the roughness of the surface, and the isotropy of the zeolite structure, no preferential orientation of the crystals was observed. It can also be observed how the small zeolite crystals are filling the porosity of the alumina support.

#### 2.2.2. Growth of ITQ-29 Zeolite on Alumina Supports

The conditions for the standard synthesis of ITQ-29 zeolite involve the use of a highly concentrated gel with low amount of water (H_2_O/SiO_2_ = 3) [24], and these were also the conditions employed in the previous preparation of ITQ-29 membrane [31]. This low water amount makes the mixture to be a very thick gel, close to a solid, that gives rise to a lower than desired interaction of the seeded support with the solid gel. Thus, the influence of the water content in the synthesis gel used for secondary growth was investigated, by changing the H_2_O/SiO_2_ ratio between 5 and 13. Also, the preparation procedure of the synthesis gel for the secondary growth was modified in order to decrease its viscosity and enhance the contact with the seeded alumina support. The detailed preparation method is described in Materials and Methods section, consisting basically on the evaporation of water beyond the desired composition, homogenization of the gel into a fine powder, and final adjustment of the water content to reach the required molar composition. This methodology allowed us to successfully obtain a viscous rather than solid synthesis gel.

**Figure 5:**
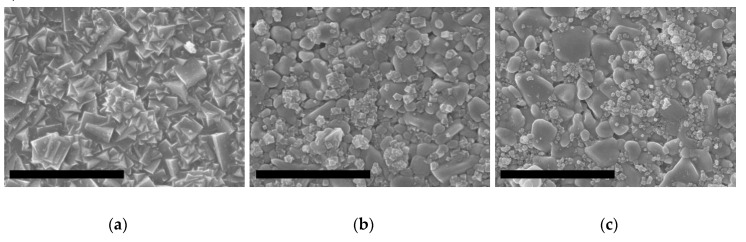
SEM images of the materials grown on the alumina supports from gels of different H_2_O/SiO_2_ molar ratios: (**a**) 5; (**b**) 7; (**c**) 13. Scale bars correspond to 30 µm.

SEM inspection of the membranes obtained using this new procedure and different H_2_O/SiO_2_ ratios is shown in Figure 5. It was found that the ITQ-29 membranes can only be obtained by using a H_2_O/SiO_2_ molar ratio of 5 so, it was selected as the proper ratio. In the case of higher water content, the ITQ-29 crystals did not form an intergrown layer on the alumina surface, and these crystals can be observed attached to the support, whereas for the preparation with H_2_O/SiO_2_ = 5 a good coverage of the alumina surface with a continuous layer of intergrown ITQ-29 cubic crystals is observed (Figure 5a).

**Figure 6:**
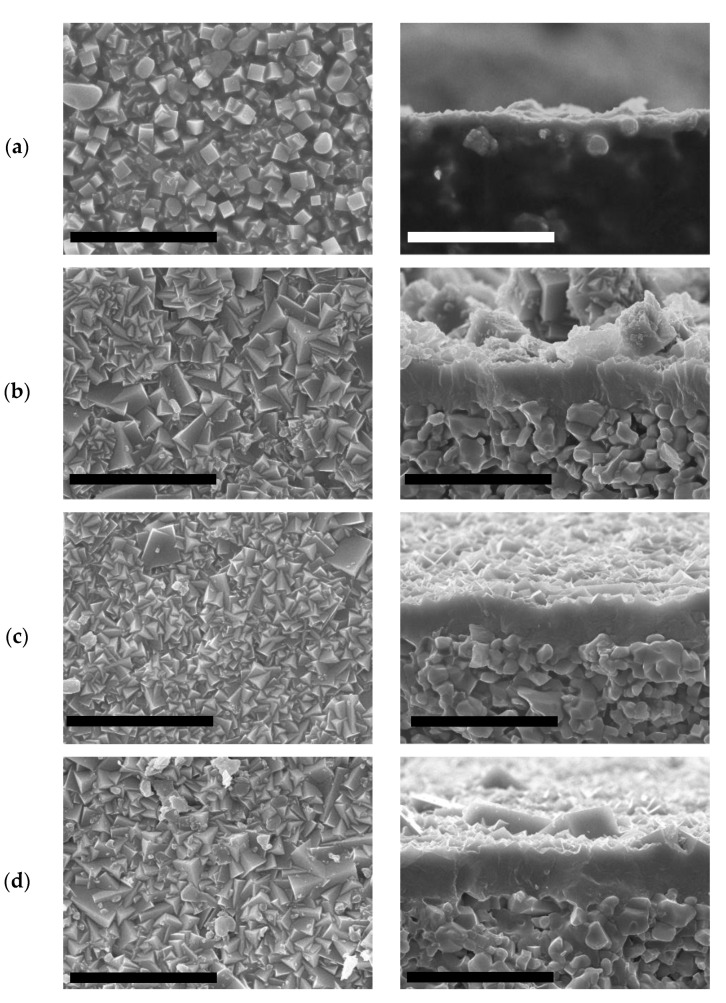
SEM images of top (left) and side (right) views of ITQ-29 materials grown on the alumina supports prepared at different crystallization times: (**a**) 3 days; (**b**) 7 days; (**c**) 10 days; (**d**) 14 days. Scale bars correspond to 30 µm.

Another key aspect on the synthesis of zeolite membranes is the duration of the secondary growth. Therefore, its influence on the membrane quality was studied herein. Both the top surface and side views of the materials prepared after 3, 7, 10, and 14 days are collected in Figure 6. In all cases the H_2_O/SiO_2_ molar ratio was 5.

**Figure 7:**
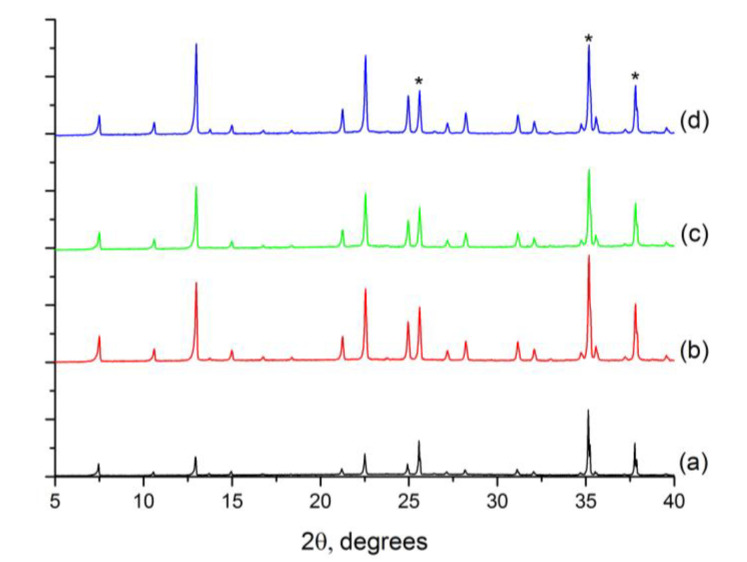
X-ray diffraction patterns of ITQ-29 materials grown on the alumina supports prepared at different crystallization times: (**a**) 3 days; (**b**) 7 days; (**c**) 10 days; (**d**) 14 days. *: alumina peaks from the support.

All materials correspond with LTA structure according to XRD analysis collected in Figure 7, although the intensities of the peaks corresponding to LTA zeolite are lower in the membrane prepared at 3 days. In this case, even if the seeds grow to some extent, the presence of an intergrown zeolite layer was not observed, and the alumina support is still visible (Figure 6a). The LTA peaks are found, arising from the crystals attached to the surface. On the other hand, continuous ITQ-29 zeolite layers were obtained when the secondary growth was extended between 7 and 14 days. After 7 days, the thickness of the membrane was 8 µm. For longer times, the thickness remains at around 10 µm after 10 and 14 days, observing that longer times do not imply a re-dissolution of the zeolite layer or competitive formation of other crystalline phases.

The occluded organic structure-directing agent (OSDA) must be removed from the zeolite to open its porosity, and calcination in air was performed at different temperatures to study its impact on the membrane quality. The calcination process is a key point in the preparation of high silica zeolite membranes since the high temperature required usually produces cracks in the zeolite layer making the membrane useless for further applications. This was one of the main problems of the previously reported ITQ-29 membrane that suffered of crack formation when calcined in standard conditions and it required the use of ozone and low temperature for partially removing the organic and open the porosity without damaging the zeolite layer [31].

**Figure 8:**
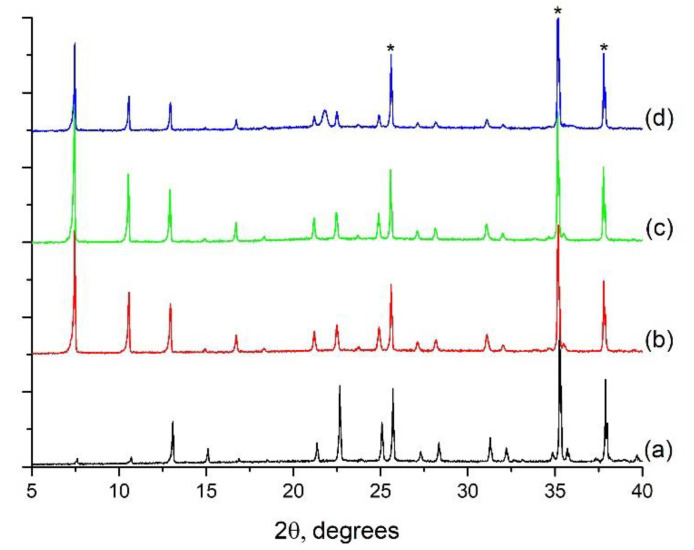
XRD patterns of pure silica ITQ-29 materials grown on the alumina supports: (**a**) uncalcined; (**b**) calcined at 773 K; (**c**) calcined at 823 K; (**d**) calcined at 873 K. *: alumina peaks from the support.

The materials were calcined at 773, 823, and 873 K, and the corresponding XRD analyses are summarized in Figure 8. The relative intensities of the three characteristic peaks of the zeolite between 6 and 13° of 2θ changed after the organic removal, being more intense in the calcined materials than in the as synthesized form, as typically occurs upon calcination. This is due to differences in the electronic density within the pores when the zeolite is empty (after calcination) or filled with organic and/or water molecules [36,37]. From this point of view, calcination at 773 K for 6 h seems to be sufficient to remove the OSDA. After calcination at 873 K, a significant loss of signal intensity was found, and a wide peak at 21.8 degrees arises, probably because of densification of the material and formation of cristobalite. The crystalline structure is preserved after calcination below this temperature.

**Figure 9:**
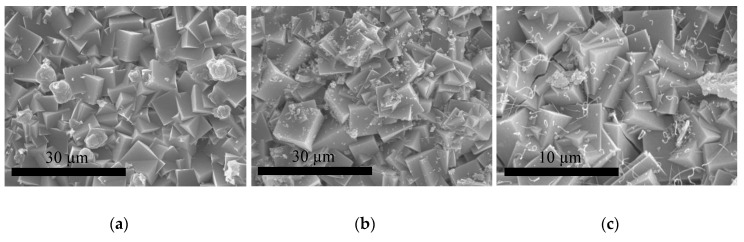
Top view SEM images of ITQ-29 materials grown on the alumina supports after calcination at: (**a**) 773 K; (**b**) 823 K; (**c**) 873 K.

The surface of the calcined materials was analyzed by SEM to confirm or discard the formation of cracks. The images are shown in Figure 9. The membrane calcined at 773 K is crack-free, but minor cracks were found after calcination at 823 K. The cracks are more abundant after calcination at 873 K. The presence of cracks would hinder their performance when used in a separation process. So, according to XRD and SEM analysis, the calcination at 773 K for 6 h was found to be sufficient for the activation of the ITQ-29 membrane, yielding a crack-free zeolite layer on the alumina support. It has to be noted that the color of the membrane calcined at 773 K was white, confirming that all the organic has been removed.

**Figure 10:**
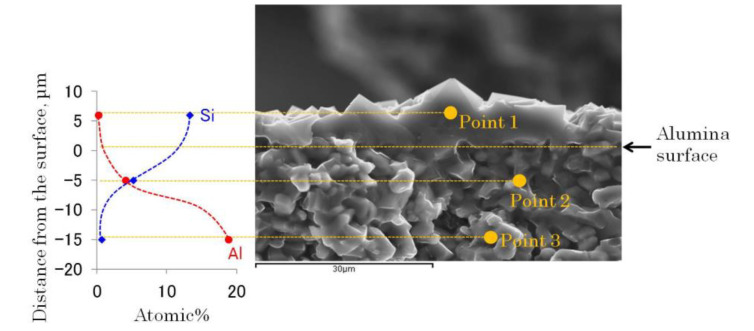
Si and Al composition along the ITQ-29 material grown on the alumina support.

The aluminum content on the zeolite has an impact on its polarity, and it is crucial to obtain pure silica membranes to avoid olefins oligomerization during the olefin/paraffin separation, and take advantage of its high hydrophobicity during water/bioalcohols separation. For that reason, energy dispersive X-ray spectroscopy (EDX) analyses were performed on the calcined pure silica ITQ-29 membrane to study if aluminum incorporation into the zeolitic framework took place in some extent, and also to analyze the zeolite intrusion into the porous support. The selected material was prepared after 7 days of secondary growth, and calcined for 6 h at 773 K. Atomic percentages of silicon and aluminum were analyzed along the membrane, and the results are shown in Figure 10. It was found that, within the detection limits, the aluminum was not incorporated into the zeolite layer. So, the membrane consisted of pure silica ITQ-29 zeolite and the aluminum from the support was not dissolved during the synthesis and incorporated into the zeolite framework; and it did not migrate from the support during calcination. EDX analysis at 5 µm underneath the support surface revealed that the zeolite grew within the support porosity, but silicon was not present at a depth of 15 µm.

In summary, these results confirm that pure silica ITQ-29 zeolitic layers were successfully grown on porous alumina supports by optimization of different parameters, such as the crystal size of the seeds, the preparation procedure, and the calcination conditions. The selection of planar alumina supports instead of tubular ones together with the modification of the synthesis procedure, resulted in the successful growth of a pure silica ITQ-29 zeolite layer on the support. In this manner, significant advances with respect to the previously reported ITQ-29 membrane are achieved. First, the presence of germanium is avoided with the consequent improvement in the stability and, in addition, the reduction of strains between the planar support and the zeolite layer together with its silica composition allows the calcination of the membrane avoiding the formation of cracks. The materials obtained consist of continuous layers of all silica LTA zeolite in which it is expected the same hydrophobic character as in the powder form and similar adsorptive properties, as long as the zeolite layer is continuous and defect-free. These materials are expected to be particularly useful for different separation processes involving olefins or bioalcohols, among others, that will be the subject of future studies.

## 3. Materials and Methods

### 3.1. Preparation of ITQ-29 Seeds

ITQ-29 zeolite seeds were synthesized from a low water content synthesis gel, according to the general procedure described in the literature [24]. The organic structure-directing agents used were 4-methyl-2,3,6,7-tetrahydro-1H,5H-pyrido [3.2.1-ij] quinolinium hydroxide (ROH) and tetramethylammonium hydroxide (TMAOH). The gel was prepared by hydrolyzing tetraethylorthosilicate (TEOS) in an aqueous solution of ROH and TMAOH. The mixture was then stirred until ethanol formed upon hydrolysis of TEOS and the excess of water were evaporated to reach the desired gel composition. After that, an aqueous solution of HF was added. Finally, previously prepared as-made ITQ-29 powder dispersed in water, was added as seed crystals, taking into account that 5 wt.% of SiO_2_ in the synthesis gel arises from these seed crystals. So, pure silica ITQ-29 was synthesized from a gel with the following composition:

SiO_2_: 0.25ROH: 0.25TMAOH: 0.5HF: 3H_2_O

The solid synthesis gel was introduced into Teflon-lined stainless steel autoclaves. For crystallization, the autoclaves were heated at 398 K for 3.5 and 4.5 days under static conditions or continuous rotation of the autoclaves at 45 r.p.m. Then, the autoclaves were cooled down, and the solids were filtered, washed with distilled water and acetone, and dried at 373 K.

### 3.2. Preparation of ITQ-29 Zeolite on Alumina Supports

Uncalcined zeolite ITQ-29 with a crystal size of 0.6 µm prepared as described above was used for seeding asymmetric α-alumina disks, with pore diameter d_50_ = 1.8 µm, 3 mm thickness, and 25 mm in diameter, from Fraunhofer (Hermsdorf, Germany).

The alumina supports were seeded by dip-coating them into an ITQ-29 zeolite dispersion in aqueous solution of polyethyleneimine (PEI, average molecular weight = 25,000) [38]. The zeolite concentration was 0.5 wt.% in water and it was previously sonicated for 1 h before adding the PEI to reach a 2 wt.% concentration in the total mixture. The adhesion of the zeolite crystals to the support is enhanced through the formation of NH_2_-OH linkage [39]. The supports were dipped into the dispersion and dried at 333 K for three times, and finally calcined at 773 K for 3 h in order to fire the polymer and promote the subsequent formation of OH bridges.

**Figure 11:**
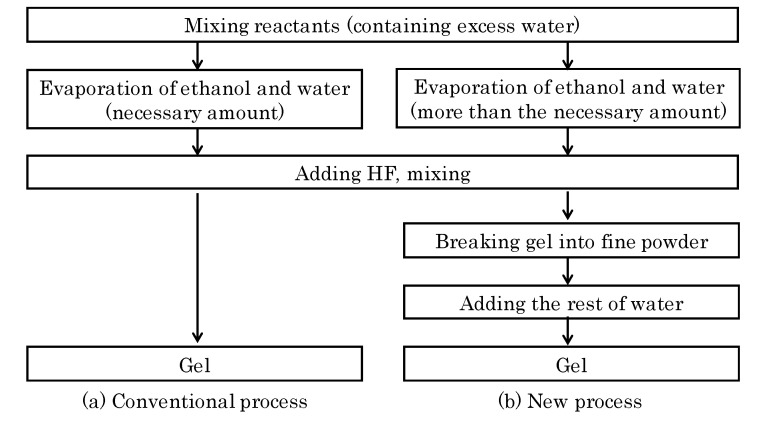
Preparation of the synthesis gel: (**a**) conventional process; (**b**) new process.

The seeded supports were then placed facing downwards in a Teflon-lined stainless steel autoclave with synthesis gels of the following molar composition:

SiO_2_: 0.25ROH: 0.25TMAOH: 0.5HF: xH_2_O

where x was modified between 5 and 13. The reactants were the same as those used for the synthesis of the seeds, but the preparation procedure of the gels for the secondary growth was modified with respect to the standard recipes in order to decrease the viscosity and improve the interaction with the seeded support. Figure 11 shows the steps for the synthesis gel preparation, where the main differences with the conventional one consisted on the evaporation of water beyond the desired composition, homogenization of the gel into a fine powder, and final adjustment of the water content to reach the chosen molar composition. This procedure resulted in a viscous rather than solid synthesis gel. The seeded supports were submitted to secondary growth at 398 K, for different durations, between 3 and 14 days, in static conditions.

The materials were finally calcined in static air at temperatures between 773 and 873 K for 6 h in order to remove the occluded organic structure-directing agent and expose the microporosity, with heating and cooling ramps of 1 K/min.

### 3.3. Characterization Tecniques

The crystallinity and phase purity of the zeolites and membranes was studied by X-ray diffraction (XRD) in a Cubix’Pro diffractometer from Panalytical (Almelo, The Netherlands), using CuKα radiation (λ_1_ = 1.5406 Å) at 45 kV and 40 mA in the 2θ range from 4 to 40°. The instrument is equipped with an X′Celerator detector and automatic divergence and reception slits (constant irradiated area of 3 mm). The affinity of the materials to water was studied by immersion calorimetry using a C80 Calvet calorimeter from Setaram (Lyon, France). The samples were degassed in a glass ampoule with a brittle bottom and were submersed in water at 303 K inside the measuring cells. Once the thermal equilibrium was reached, the ampoule was pushed down and the brittle end was broken, thus allowing the entrance of water in the ampoule wetting the sample. The heat flow was recorded as a function of time, and the heat of immersion can be calculated as the area underneath the peak, in J/g. Scanning electron microscopy (SEM) was used to study the crystal morphology and size of the ITQ-29 powder on the one hand, and the membrane topology and thickness on the other. The membranes were broken by fracture and mounted vertically on a SEM sample holder for the side view inspection. The elemental composition of the membranes was analyzed by energy dispersive X-ray spectroscopy (EDX), allowing to investigate the silicon intrusion within the alumina support and the aluminum incorporation onto the zeolite framework. The SEM and EDX analysis was done in a Jeol JSM 5410 microscope (Jeol, Tokyo, Japan) of the Electron Microscopy Service of the Universitat Politècnica de València.

## 4. Conclusions

Pure silica ITQ-29 zeolite layers were successfully synthesized on porous alumina disks by selecting the appropriate combination of crystal size of the seeds, the seeding technique, the methodology for the preparation of the gel, the duration of the secondary growth, and the calcination conditions. The water content of the synthesis gel was adjusted after the silica precipitation, hence resulting in a viscous gel rather than a solid material, thus favoring a better contact with the seeded support. In this way, continuous layers of 8 µm thickness were prepared after 7 days of secondary growth, and were activated by calcination at 773 K in air, resulting in crack-free microporous membranes. The incorporation of aluminum from the support into the zeolite layer did not occur, neither during the secondary growth nor through migration of aluminum species during calcination. The materials obtained were defect-free and crystalline, being appropriate candidates for bioalcohols separation in aqueous solutions.
